# The association of *MEFV* gene mutations with the disease risk and severity of systemic juvenile idiopathic arthritis

**DOI:** 10.1186/s12969-020-00427-8

**Published:** 2020-05-12

**Authors:** Linqing Zhong, Wei Wang, Ji Li, Mingsheng Ma, Lijuan Gou, Changyan Wang, Zhongxun Yu, Tiannan Zhang, Yanqing Dong, Qijiao Wei, Hongmei Song

**Affiliations:** Department of Pediatrics, Peking Union Medical College Hospital, Chinese Academy of Medical Sciences and Peking Union Medical College, No 1, Shuaifuyuan, Dongcheng District, Beijing, 100730 China

**Keywords:** *MEFV* gene, Systemic juvenile idiopathic arthritis, Disease risk, Disease severity

## Abstract

**Background:**

Systemic juvenile idiopathic arthritis (sJIA) has many clinical features overlapping with familial Mediterranean fever (FMF), which is caused by mutations in *MEFV* gene. And FMF patients were easily misdiagnosed as sJIA in China. So we speculate that *MEFV* is critical genetic background for sJIA and influences patients’ severity. In this study, we aim to figure out whether *MEFV* mutations are risk factor for the occurrence of sJIA and to study the association of *MEFV* mutations with disease severity of sJIA patients.

**Methods:**

The present study includes 57 sJIA children and 2573 healthy controls. Odd ratio with 95% confidence interval based on allelic frequency of *MEFV* mutations or variants was used to evaluate their contribution to sJIA susceptibility. Meta-analysis was then performed to reach comprehensive conclusion. All included sJIA patients were grouped by presence and number of *MEFV* mutations. Clinical data and indicators of disease severity were compared among different groups. Multiple linear regression method was used to find out whether the number of *MEFV* variants is associated with the severity of sJIA. Kaplan-Meier curves and log rank test were used to estimate the probability of the first relapse.

**Results:**

The *MEFV* mutations of our subjects predominantly existed in exons 2 and 3. No significant difference was found in allelic frequency between sJIA children and healthy controls. Meta-analysis demonstrated that p.M694V/I was a risk factor for sJIA (pooled OR: 7.13, 95% CI: 3.01–16.89). The relative period of activity was significantly lower in the one mutation group than those with more than one mutation (*p* = 0.0194). However, no relevance was found in multiple linear regression models.

**Conclusions:**

The mutation p.M694V/I in *MEFV* might be a risk factor for sJIA. SJIA patients carrying more than one heterozygous mutation in *MEFV* tend to be more severe than those containing only one, but studies in other cohort of patients need to be performed to validate it.

## Introduction

It’s widely believed that dysregulation of the innate immune system is the important pathogenesis for systemic juvenile idiopathic arthritis (sJIA), especially the overexpression of pro-inflammatory cytokines, such as IL-1β and IL-18 [1, 2], whose maturation and secretion primarily depend on inflammasome. SJIA does have several features overlapping with inflammasome diseases because of the partially overlapped mechanism, which are confusing for physicians when making differential diagnosis. For instance, familial Mediterranean fever (FMF), an inflammasome disease of autosomal recessive inheritance, is an important differential diagnosis from sJIA.

FMF cases were most reported in races living in Mediterranean region and are characterized by recurrent episodes of fever and serositis. However, manifestations of FMF patients in China are not so typical. For example, some FMF patients presented as recurrent fever exclusively and duration of each episode was variable, from 1 day to a week. These patients were easily misdiagnosed as sJIA in the past. With the development of sequencing technology, homozygous or compound heterozygous pathogenic variants or variants of uncertain significance (VUSs) in *MEFV* were found. Moreover, these patients responded well to colchicine. As a result, they are generally considered as FMF patients in China. Lack of recognition was another significant cause leading to misdiagnosis.

We combined the clinical criteria [3] with sequencing results of *MEFV* to confirm the diagnosis of FMF, thus to avoid misdiagnosis in clinic. Intriguingly, when we made differential diagnoses, we discovered that many sJIA patients carry heterozygous mutations of *MEFV*, most in the exon 2 and 3. In view of these facts, we speculate that *MEFV* is critical genetic background for sJIA and influences patients’ severity. Therefore, in the present study, we sequenced *MEFV* in children diagnosed with sJIA, and compared the frequency of each mutation with those in healthy controls, followed by meta-analysis, to comprehensively find out whether *MEFV* mutations contributed to the occurrence of sJIA. We further studied the association of *MEFV* mutations with sJIA patients’ severity.

## Materials and methods

### Study population

**Fig. 1:**
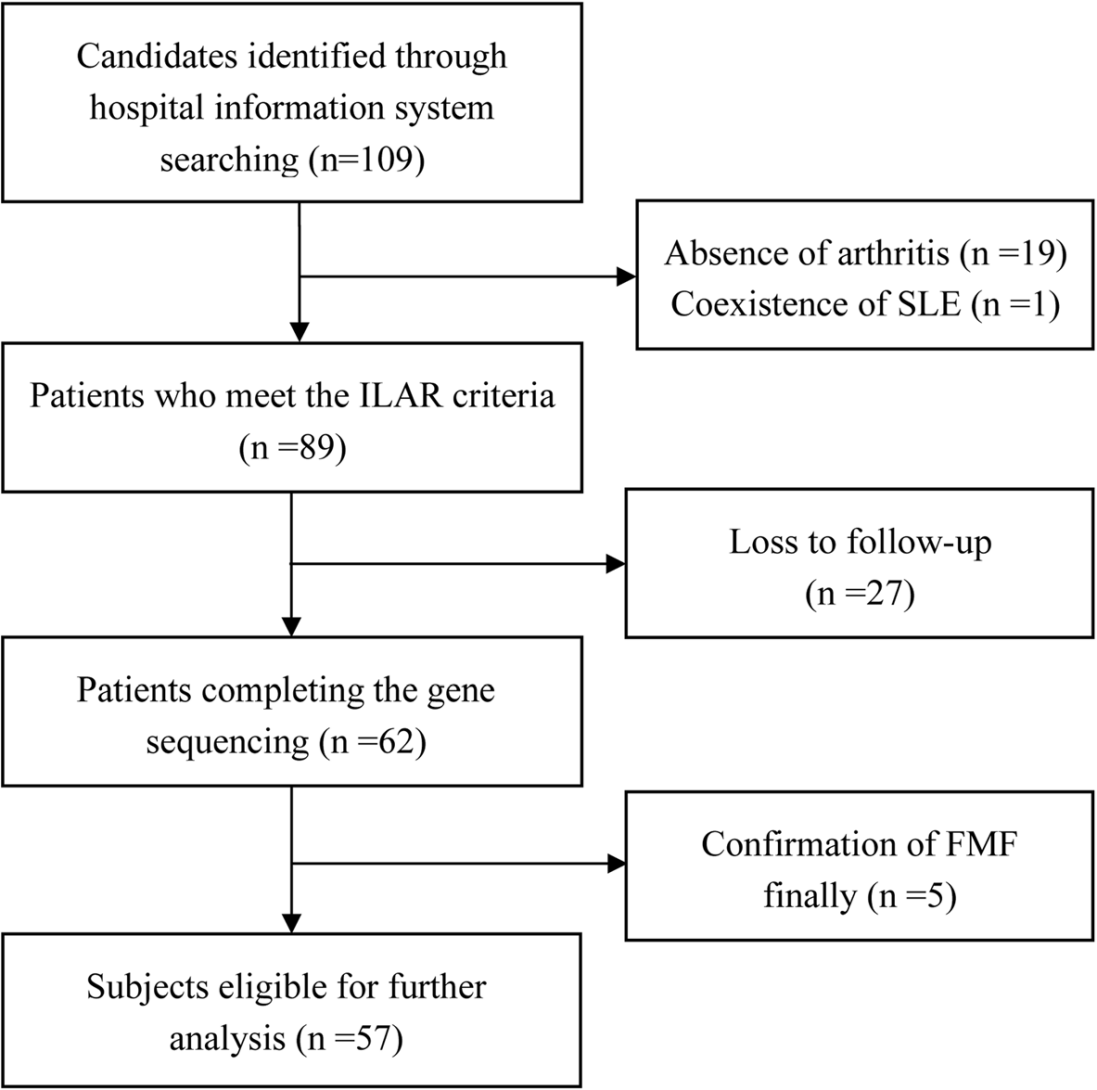
Flow diagram for the selection of the 57 eligible subjects in the study

A total of 109 children suspected with sJIA were recruited consecutively for selection between January 2011 and August 2018. Finally, a total of 57 patients were eligible for further risk and severity analysis. The flow diagram for the selection process are showed in Fig. 1. All children meet the ILAR criteria [4] and were followed up for a minimum period of 6 months in Peking Union Medical College Hospital. Those who met the following items were excluded: 1) Patients with other etiological factors such as infections, malignancy, other rheumatic diseases and so on; 2) Those who disagreed to participate in the study or lost to follow up; 3) Patients who not only met Tel Hashomer criteria [3], but also had homozygous/compound heterozygous pathogenic mutations or variants of uncertain significance (VUSs) in *MEFV*, according to the new workflow of International Study Group for Systemic Autoinflammatory Diseases (INSAID) [5], were diagnosed with FMF and were then excluded. *MEFV* gene data of healthy controls was obtained from the inner database in Beijing Novogene Bioinformatics Technology Co., Ltd., which included whole exon sequencing results of 2573 asymptomatic employees. And all healthy controls have neither family history of autoimmune nor autoinflammatory disorders. The protocol of this study was approved by the Local Ethics Committee of the Peking Union Medical College Hospital (Protocol number: ZS-1465), and written informed consent was obtained from the parents of the participants prior to the study. The healthy controls signed informed consents form to their company and the sequencing data is open to the researchers who worked with them.

### *MEFV* mutation sequencing

A 2 mL venous blood sample was collected in a sterile EDTA vacutainer. Genomic DNA was prepared according to manufacturer’s protocols (Rapid Blood DNA Kit, Biomed, China). Whole exon sequencing was applied to most cases and all healthy controls. Each sample was enriched for sequencing according to the Illumina protocols. The amplified DNA was sequenced on a HiSeq 2000 sequencer (Illumina, San Diego, CA). Gene panel was applied to four cases. Briefly, a specific primary immunodeficiency disease panel was used based on targeted exome capture technology. The gene lists of this panel are showed in the Table S1 of Online Resource 1. The results were verified by Sanger sequencing, of which the information of primers is presented in the Table S2 of Online Resource 1.

### Collection of clinical data

All clinical data was extracted from inpatient or outpatient medical records. Case report forms were designed before data collection, which included information about demographic and clinical data and were filled in by paediatric rheumatological physicians. Laboratory results before treatment were accessed for further analysis.

### Grouping

When we evaluated the association of *MEFV* mutations with disease severity, all included sJIA patients were first divided according to whether they have *MEFV* mutations or not. Those with *MEFV* mutation(s) were further separated into two groups, namely, patients with one mutation and those with more than one mutation in *MEFV*. We didn’t group sJIA patients according to type of mutation because their genotypes were variable. Demographic data, clinical characteristics, as well as indicators of disease severity were compared among these three groups.

Disease severity was objectively measured with the following items. (1) average duration of use of each drug, i.e., the ratio of the length of follow-up (months) to the number of drugs administered. Medications for sJIA children were generally adjusted based on a step-up program in our department, according to the American College of Rheumatology Recommendations [6, 7]. Therefore, average duration of administration of each drug reflected disease refractory to some extent. The shorter each drug is used, the more frequent it is to change treatment regimens, saying that it’s more difficult to control the systemic inflammation. (2) the relative period of activity, i.e., the proportion of disease activity duration to total follow-up period (months). (3) relative relapse rate, i.e., the ratio of frequency of relapse to the length of follow-up (months). Remission was defined as clinical inactive diseases according to American College of Rheumatology Criteria [8].

### Statistical analysis

Quantitative data are presented as mean (S.D.), or median (IQR) in case of skewed data, while qualitative data are presented as frequency and proportion. When comparing data between two groups, the Student’s *t*-test or Mann-Whitney U test were applied to calculate intergroup differences for continuous variable. Comparisons among three or more subgroups were performed with analysis of variance or Kruskal-Wallis test. The Pearson chi-square test or Fisher’s exact test was employed in terms of categorical variable.

The association between *MEFV* mutations and risk of sJIA was assessed with odds ratios (ORs) and 95% confidence intervals (95% CI) based on allelic frequency. To obtain comprehensive conclusions, meta-analysis was further performed to find out whether *MEFV* mutations contributed to the occurrence of sJIA. The detailed process of meta-analysis was presented in the last paragragh of Online Resource 1.

To find out whether the number of *MEFV* variants is associated with the severity of sJIA, multiple linear regression method was used. Variables that were considered clinically relevant or that showed a univariate relationship with outcome went into the multiple linear regression models. Considering the sample size of this study, we limited the number of variables to six; the maximum number of variables is 5 to 10% of the sample size, to ensure parsimony of the final models. A two-sided *P* value less than 0.05 was considered statistically significant. Kaplan-Meier curves and log rank test were used to estimate the probability of the first relapse. All analyses were performed using SPSS version 19.0.0 (IBM Corp., Armonk, NY) and Graphpad Prism version 7 software (La Jolla, CA, USA).

## Results

### Demographic and clinical characteristics of patients

A total of 109 children suspected with sJIA were recruited consecutively for selection between January 2011 and August 2018. Eighty-nine patients were eligible according to the ILAR criteria [4], while 19 were excluded for absence of arthritis even though followed-up for several years, and one child was ruled out for complication of systemic lupus erythematosus 2.5 years after the onset of sJIA. Twenty-seven patients’ blood samples were unavailable for loss to follow up, thus *MEFV* gene sequencing was finished in 62 patients. Then five children were excluded from further analysis, because homozygous/compound heterozygous pathogenic mutations or VUSs in *MEFV* were found and then diagnosis of FMF was confirmed after reviewing their medical history. At last, a total of 57 patients were eligible for further risk and severity analysis. Their demographic and clinical characteristics are presented in Table 1.

**Table 1:** Demographic and clinical characteristics of patients eligible for current study

	Patients included in current study (*n* = 57)
Onset age, median (IQR), years	6.42 (5.30)
Male, n (%)	28 (49.1%)
Time to initiation of therapy, median (IQR), months	1.00 (1.00)
Follow-up period, median (IQR), months	54.00 (75.00)
Number of the affected joints, median (IQR)	5 (11)
**Manifestations, n (%)**	
Fever	57 (100%)
Skin Rash	50 (87.7%)
Joint Swelling or arthralgia	57 (100%)
Hepatomegaly	16 (28.1%)
Splenomegaly	24 (42.1%)
Lymphopathy	30 (52.6%)
Serositis	13 (22.8%)
MAS	6 (10.5%)
**Lab examinnation**^a^	
ESR (mm/h)	78.77 (30.63)
CRP (mg/L)	130.00 (109.83)
SF (ng/ml)	1376.00(2670.00)
WBC (×10^9/L)	16.14 (11.91)
PLT (×10^9/L)	444 (194)

*Abbreviation*: *IQR* interquartile range, *MAS* macrophage activation syndrome, *ESR* erythrocyte sedimentation rate, *CRP* C reactive protein, *SF* serum Ferritin, *WBC* white blood cells; *PLT* platelets

^a^The data of ESR is represented in mean(S.D.), while other laboratory items in median (IQR)

### The allelic frequency of *MEFV* mutations and their association with the risk of sJIA

The sequencing results showed that mutations or variants of *MEFV* in Chinese sJIA patients primarily existed in exons 2 and 3. The most common variant was E148Q (22.8%), all of which were heterozygous, followed by c.1759 + 8c > t (7.0%) and R202Q (5.3%). Likewise, E148Q was most frequent in the healthy controls, with a proportion of 25.8%. Moreover, homozygous genotype of E148Q was found in 6.3% of the healthy population.

To figure out whether *MEFV* mutations contributed to the occurrence of sJIA, the allelic frequency of eligible patients and the 2573 healthy controls was then compared by calculating the ORs with 95% CIs, which revealed no significant difference between our group of Chinese sJIA children and the healthy population, as we can see in Table 2.

**Table 2:** The Allelic Frequency of *MEFV* mutations or variants in the current study

Mutation or variants	sJIA patients(*n* = 57)		Controls(*n* = 2573)		OR	95%CI
	mutated allele	wide type allele	mutated allele	wide type allele		
E148Q	26	88	1326	3820	0.85	0.55–1.32
L110P	4	110	337	4809	0.52	0.19–1.42
R202Q	6	108	191	4955	1.44	0.63–3.32
G304R	1	113	70	5076	0.64	0.09–4.66
P369S	5	109	356	4790	0.62	0.25–1.52
R408Q	4	110	249	4897	0.72	0.26–1.96
c.1759 + 8c > t	8	106	521	4625	0.67	0.32–1.38

R202Q, G304R, and c.1759 + 8c > t were regarded as benign or likely benign variants while other variants in the table were categorized as variants of uncertain significance according to the new workflow of International Study Group for Systemic Autoinflammatory Diseases

*Abbreviation*: *sJIA* systemic juvenile idiopathic arthritis

**Fig. 2:**
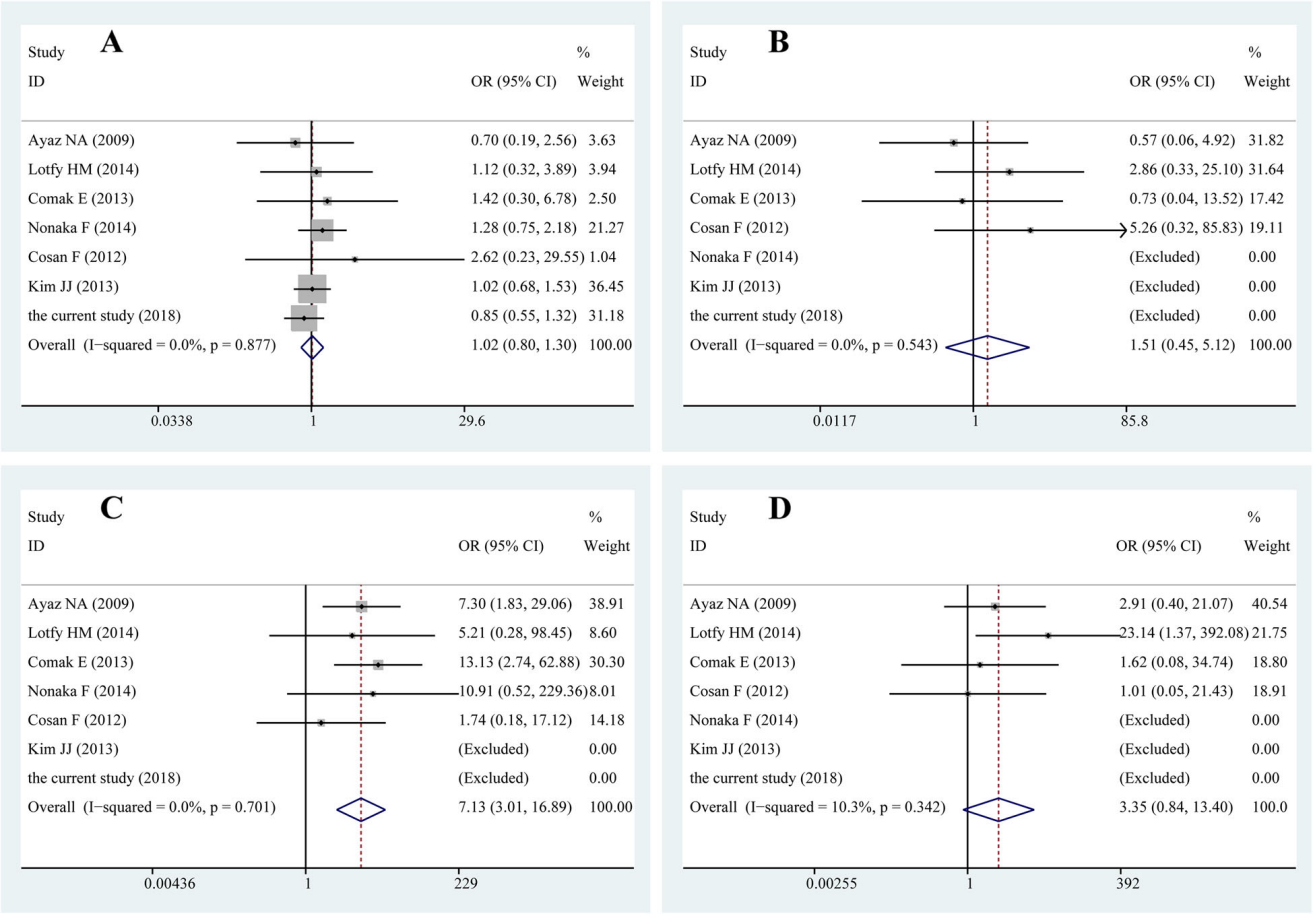
Forest plot of the association of *MEFV* mutations with sJIA susceptibility. **a** E148Q; **b** M680I; **c** M694V/I; **d** V726A

In order to draw more comprehensive conclusions, pooled OR and corresponding 95% CI was further calculated through meta-analysis. Seven studies (include the current study) have explored the association of *MEFV* mutations and the risk of sJIA [9–14] and were therefore included in the meta-analysis, among which three were about adult onset still’s disease (AOSD). Because sJIA and AOSD were considered as a continuum of a single disease entity as for similar pathogenesis [15], genetic background [16] and manifestations, patients of both diseases were included in the final meta-analysis. General characteristics of eligible studies were listed in the Table S3 of the Online Resource 2. The most widely studied mutations are E148Q, M680I, M694V/I and V726A. However, in Asian studies [12, 14], mutations in exon 10 were seldom and M694V heterozygous mutation was only found in two AOSD patients in Nonaka’s study [12]. M680I and V726A were not discovered both in sJIA patients and healthy controls, including the present study. Forest plots of the meta-analysis were presented in Fig. 2. The quantitative meta-analysis demonstrated that M694V/I was more prevalent in sJIA/AOSD patients than healthy controls (pooled OR: 7.13, 95% CI: 3.01–16.89, p<0.001, see Table S4 in Online Resource 2). Potential publication bias was evaluated with Begg’s funnel plots and Egger’s test, both of which suggested the absence of publication bias (Begg’s funnel plots refer to Online Resource 2, and *p* values of egger’s test were 0.542, 0.823, 0.568, and 0.947 respectively).

### The relevance between disease severity and the number of *MEFV* mutations

The 57 sJIA children who underwent *MEFV* sequencing were divided into three groups according to the number of mutations, namely, patients without mutation, with one mutation and those with more than one mutation in *MEFV*. Several aspects were firstly compared among these groups, including demographic characteristics, manifestations, laboratory results, as well as treatments, as showed in Table 3. Prevalence of biologics use was significantly lower in the one mutation group than the no mutation group after pairwise comparison. There was no prominent difference found among the three groups in prevalence of biologics use when patients who had not adhered to treatments excluded (*p* = 0.054).

**Table 3:** Characteristics of patients stratified by the presence and number of *MEFV* mutations

	Patients without MEFV mutation (*n* = 28)	Patients with one heterozygous mutations (*n* = 18)	Patients with more than one heterozygous mutations (*n* = 11)	*P* value
Onset age, median (IQR), years	6.09 (4.36)	7.33 (5.27)	5.33 (6.50)	0.325
Male, n (%)	10 (35.7%)	12 (66.7%)	6 (54.5%)	0.121
Time to initiation of therapy, median (IQR), months	1.00 (1.00)	1.25 (2.25)	2.00 (2.00)	0.256
Number of the affected joints, median (IQR)	5 (13)	5 (6)	11 (18)	0.262
**Manifestations, n (%)**				
Fever	28 (100%)	18 (100%)	11 (100%)	–
Skin Rash	25 (89.3%)	15 (83.3%)	10 (90.9%)	0.870
Joint Swelling or arthralgia	28 (100%)	18 (100%)	11 (100%)	–
Hepatomegaly	10 (35.7%)	4 (22.2%)	2 (18.2%)	0.538
Splenomegaly	15 (53.6%)	4 (22.2%)	5 (45.5%)	0.109
Lymphopathy	15 (53.6%)	9 (50.0%)	6 (54.5%)	1.000
Serositis	7 (25.0%)	4 (22.2%)	2 (18.2%)	1.000
MAS	3 (10.7%)	1 (5.6%)	2 (18.2%)	0.643
**Lab examinnation**^a^				
ESR (mm/h)	76.36(29.62)	80.06(34.81)	82.82(28.08)	0.825
CRP (mg/L)	132.00(112.00)	140.00(119.50)	72.61(83.00)	0.182
SF (ng/ml)	1832.00(3848.00)	1361.00(1425.00)	881.00(1329.00)	0.184
WBC (×10^9/L)	17.20(12.66)	14.80(11.09)	16.44(5.75)	0.860
PLT (×10^9/L)	452(134)	462(156)	463(136)	0.960
**Treatment, n (%)**				
GC	27 (96.4%)	18 (100%)	10 (90.9%)	0.447
MTX	27 (96.4%)	18 (100%)	10 (90.9%)	0.447
NSAIDs	26 (92.9%)	16 (88.9%)	11 (100%)	0.661
HCQ	14 (50.0%)	9 (50.0%)	3 (27.3%)	0.444
LEF	14 (50%)	8 (44.4%)	5 (45.5%)	0.940
CsA	13 (46.4%)	8 (44.4%)	5 (45.5%)	1.000
Biologics	23 (82.1%)^b^	8 (44.4%)^b^	8 (72.7%)	0.025

*Abbreviation*: *IQR* interquartile range, *MAS* macrophage activation syndrome, *ESR* erythrocyte sedimentation rate, *CRP* C reactive protein, *SF* serum ferritin, *WBC* white blood cells, *PLT* platelets, *GC* glucocorticoid, *MTX* methotrexate, *NSAIDs* non-steroid anti-inflammatory drugs, *HCQ* hydroxychloroquine, *LEF* leflunomide, *CsA* cyclosporin a

^a^The data of ESR and PLT are represented in mean(S.D.), while other laboratory items in median (IQR)

^b^Significance was found after pairwise comparison with Bofferoni correction

**Fig. 3:**
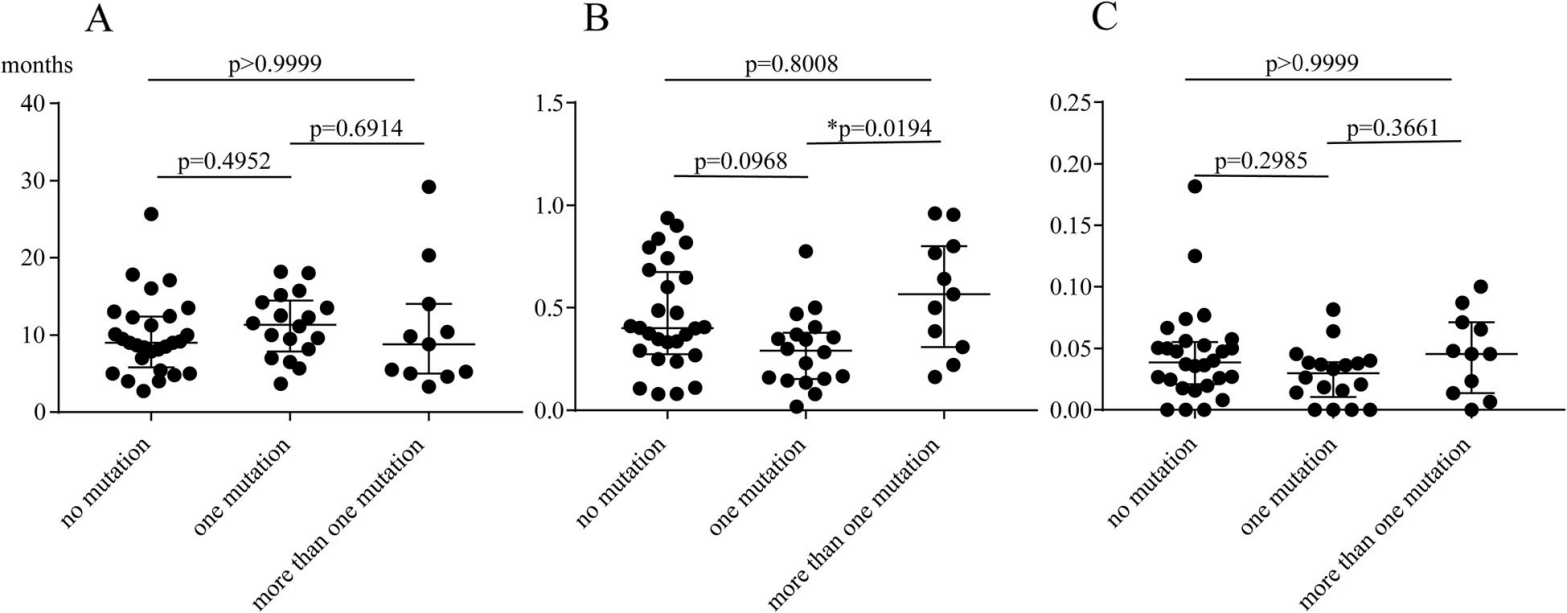
Comparison of **a** average duration of use of each drug, **b** the relative period of activity, **c** relative relapse rate among the no mutation group, the one mutation group and the more than one mutation group

Next, disease severity was compared among the above three groups. The indicators of disease severity involved in this study were average duration of use of each drug, the relative period of activity and relative relapse rate, as described in the methods section. Significant difference was not discovered in the average duration of use of each drug and the relative relapse rate. On the contrary, the relative period of activity was significantly lower in the one mutation group than the more than one mutation group (Fig. 3). The same was true when patients who had not adhered to treatments ruled out (*p* = 0.002).

**Fig. 4:**
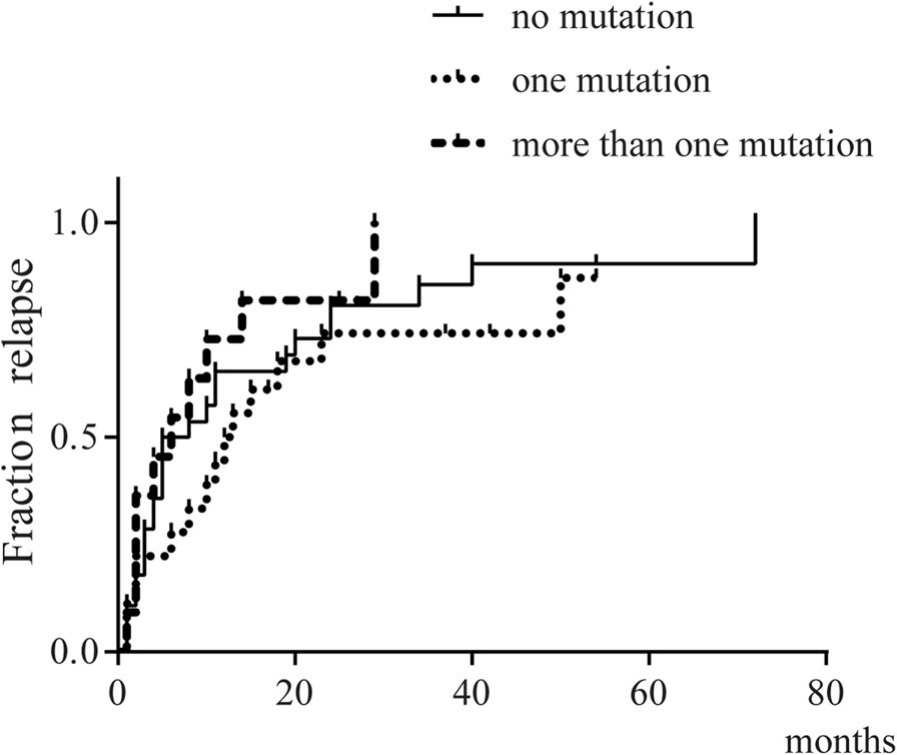
Kaplan-Meier curves concerning the probability of the first relapse

Multiple linear regression was then performed. Variables that were considered clinically relevant or that showed a univariate relationship with outcome went into the multiple linear regression models. For the analysis of average duration of use of each drug, onset age, ESR, hepatomegaly, serositis and the number of *MEFV* variants went into the multiple linear regression model (*p* value was 0.038, 0.024, 0.135, 0.156 and 0.453 respectively). For the analysis of the relative period of activity, onset age, time to initiation of therapy, number of the affected joints, splenomegaly, serositis as well as the number of *MEFV* variants went into the multiple linear regression model (p value was 0.192, 0.103, 0.0.006, 0.195, 0.048 and 0.060 respectively). However, no relevance was found in multiple linear regression models (both *p* values are more than 0.05, data not shown). Lastly, the probability of the first relapse was estimated using Kaplan-Meier curves and log-rank test, which revealed no significant difference among the three groups (*p* = 0.3505, Fig. 4).

Association of other genes with the risk of sJIA and disease severity was not discovered (data not shown and no intention to publish them in other articles).

## Discussion

SJIA has been known for quite a long time, but its pathogenesis remains unclear yet regardless of intense study. Although the pathogenesis of sJIA is poorly understood, it’s widely believed that dysregulation of innate pro- and anti-inflammatory cytokines is important pathogenesis for sJIA, especially overexpression of pro-inflammatory cytokines, such as IL-1β, IL-6 and IL-18 [1, 2, 17]. As for the early onset age of sJIA, more and more attention was paid to the genetic background responsible for the occurrence of sJIA, especially genes of molecules involving in the above inflammatory pathways [18, 19], such as *NLRP3* [20] and TNFRSF1A [21]. Up to now whether the gene mutations have a role on the onset or the severity of sJIA remains a mystery. In the present study, we aimed to investigate the effect of *MEFV* gene mutations on the occurrence and severity of sJIA.

Fifty-seven sJIA children completing *MEFV* sequencing were eligible in our study. There was no significant difference between allelic frequency of *MEFV* mutations in our group of Chinese sJIA children and healthy controls. The mutations of *MEFV* in Chinese sJIA patients primarily existed in exons 2 and 3, which was consistent with the results in other Asian populations [12, 14]. Mutations in exon 10 were seldom found in Asian sJIA children and M694V heterozygous mutation was only found in two AOSD patients in Nonaka’s study [12]. Other widely studied mutations like M680I and V726A were not discovered both in sJIA patients and healthy controls in the present study. In fact, mutations of *MEFV* found in Asian populations were predominantly variants of uncertain significance, among which E148Q was the most common. The pathogenicity of E148Q has been always controversial. In the healthy controls, even 6.3% presented with homozygous type of E148Q. Taken the above together, we believed that these variants of uncertain significance played limited role on at least the disease onset of sJIA patients in Chinese.

To reach comprehensive conclusions, we further evaluated the contribution of *MEFV* mutations to the susceptibility of sJIA by meta-analysis. So far seven studies (including the present study) have explored the association of *MEFV* and risk of sJIA [9–14]. The most widely studied mutations are E148Q, M680I, M694V/I and V726A. Further Quantitative meta-analysis demonstrated that M694V/I was a risk factor for sJIA/AOSD. Although this potential relationship has been found, a causal relationship cannot be confirmed. That is, it is uncertain if the mutations in *MEFV* gene lead to sJIA or if this is a different FMF clinical phenotype, because homozygote or heterozygote mutations in MEFV genes can cause a clinical FMF phenotype. What’s more, four out of five studies containing M694V/I were conducted in subjects from countries around Mediterranean. Therefore, we should be cautious to extend the conclusions to other populations. More relevant investigations from other countries are favourable to reach comprehensive conclusions, and more attention should be paid to those mutations common in Asian. No relevance was found between other mutations and sJIA/AOSD. Apart from sJIA, genome-wide significant associations of MEFV *M694V* with other complex autoinflammatory diseases, such as Behςet’s disease [22] and ankylosing spondylitis [23, 24], were also discovered.

To analyse the relevance of *MEFV* mutations with disease severity in sJIA patients, we separated the 57 children into three groups according to the number of *MEFV* mutations, namely, the no mutation group, the one mutation group and the more than one mutation group. To our knowledge, it’s the first report to investigate the effect of *MEFV* mutations on sJIA disease severity. After comparing the indicators of disease severity defined in advance, we found that the relative period of activity was significantly lower in the one mutation group than the more than one mutation group. The conclusion was the same after patients who didn’t comply with treatment removed. However, no relevance was found in multiple linear regression models. Such conclusion might be attributed to the small sample of our study. In this regard, our conclusions may serve as a clue for further investigation into the role of *MEFV* mutations on sJIA disease severity.

Obviously, there are several limitations concerning our study. As an observational study, the results in our study might be influenced by some potential factors, such as doctor’s decisions, patients’ compliance, as well as other confounding factors. Small sample was another limitation, not only due to rarity of sJIA, but also because of those loss to follow-up. Therefore, more relevant studies are required to draw comprehensive conclusions in the future.

## Conclusions

In summary, this study suggests that the mutation p.M694V/I in *MEFV* gene might be a risk factor for sJIA/AOSD. Furthermore, we found that sJIA patients from China carrying more than one heterozygous mutation in MEFV tend to be more severe than those containing only one, but studies in other cohort of patients need to be performed to validate it.

## Supplementary information

**Additional file 1.** Supplemental methods.

**Additional file 2.** Supplemental results.

**Additional file 3.** Raw data of the sequencing results.

## Data Availability

All data generated or analysed during this study are included in this published article and its supplementary information files.

## Abbreviations

sJIA: systemic juvenile idiopathic arthritis
FMF: familial Mediterranean fever
VUSs: variants of uncertain significance
INSAID: International Study Group for Systemic Autoinflammatory Diseases
NGS: next generation sequencing
AOSD: adult onset still’s disease.
