# Supplementary material for: The association of MEFV gene mutations with the disease risk and severity of systemic juvenile idiopathic arthritis
Source: Pediatr Rheumatol Online J. 2020 May 12;18:38. doi: 10.1186/s12969-020-00427-8 (PMC7218505; doi:10.1186/s12969-020-00427-8)
Supplement: Supplementary file 1 — Additional file 1. Supplemental methods. [file 12969_2020_427_MOESM1_ESM.docx]

**Table S1.The list of genes captured in the primary immunodeficiency diseases panel**

| ACP5 | ACTB | ADA | ADAM17 | ADAR | AICDA | AIRE | AK2 | AP3B1 | APOL1 | ATM | B2M |
| --- | --- | --- | --- | --- | --- | --- | --- | --- | --- | --- | --- |
| BLM | BLNK | BLOC1S6 | BTK | C1QA | C1QB | C1QC | C1R | C1S | C2 | C3 | C5 |
| C6 | C7 | C8A | C8B | C8G | C9 | CARD11 | CARD14 | CARD9 | CASP10 | CASP8 | CCBE1 |
| CD19 | CD247 | CD27 | CD3D | CD3E | CD3G | CD40 | CD40LG | CD46 | CD59 | CD79A | CD79B |
| CD81 | CD8A | CEBPE | CECR1 | CFB | CFD | CFH | CFHR1 | CFHR2 | CFHR3 | CFHR4 | CFHR5 |
| CFI | CFP | CHD7 | CIITA | CLEC7A | CLPB | COL7A1 | COLEC11 | COPA | CORO1A | CR2 | CSF2RA |
| CSF3R | CTLA4 | CTPS1 | CTSC | CXCR4 | CYBA | CYBB | DCLRE1B | DCLRE1C | DKC1 | DNMT3B | DOCK2 |
| DOCK8 | ELANE | EPG5 | FADD | FAS | FASLG | FCGR3A | FCN3 | FERMT1 | FERMT3 | FOXN1 | FOXP3 |
| FPR1 | G6PC3 | GATA2 | GFI1 | GUCY2C | HAX1 | HPS1 | HPS4 | HPS6 | ICOS | IFIH1 | IFNG |
| IFNGR1 | IFNGR2 | IGLL1 | IKBKB | ZBTB24 | IKZF1 | IL10 | IL10RA | IL10RB | IL12B | IL12RB1 | IL17F |
| IL17RA | IL17RC | IL1RN | IL21 | IL21R | IL2RA | IL2RG | IL36RN | IL6 | IL7R | INO80 | IRAK4 |
| IRF7 | IRF8 | ISG15 | ITCH | ITGB2 | ITK | JAGN1 | JAK3 | KRAS | LAMTOR2 | LCK | LIG4 |
| LPIN2 | LRBA | LYST | MAGT1 | MALT1 | MAP3K14 | MASP1 | MASP2 | MCM4 | MEFV | MOGS | MRE11A |
| MS4A1 | MSH6 | MTHFD1 | MVK | MYD88 | NBN | NCF1 | NCF2 | NCF4 | NFAT5 | NFKB2 | NFKBIA |
| NHP2 | NLRC4 | NLRP12 | NLRP3 | NOD2 | NOP10 | NRAS | ORAI1 | PARN | PIK3CD | PIK3R1 | PLCG2 |
| PMS2 | PNP | POLE | PRF1 | PRKCD | PRKDC | PSMB8 | PSTPIP1 | PTPRC | RAB27A | RAC2 | RAG1 |
| RAG2 | RBCK1 | RFX5 | RFXANK | RFXAP | RHOH | RNASEH2A | RNASEH2B | RNASEH2C | RNF168 | RNF31 | RORC |
| RPSA | RTEL1 | SAMHD1 | SBDS | SEMA3E | SERPING1 | SH2D1A | SH3BP2 | SKIV2L | SLC29A3 | SLC35C1 | SLC37A4 |
| SLC46A1 | SMARCAL1 | SP110 | SPINK5 | STAT1 | STAT2 | STAT3 | STAT5B | STIM1 | STK4 | STX11 | STXBP2 |
| TAP1 | TAP2 | TAPBP | TAZ | TBK1 | TBX1 | TCF3 | TCN2 | TERT | THBD | TICAM1 | TINF2 |
| TLR3 | TMC6 | TMC8 | TMEM173 | TNFRSF13B | TNFRSF13C | TNFRSF1A | TNFRSF4 | TNFSF12 | TPP1 | TPP2 | TRAF3 |
| TRAF3IP2 | TREX1 | TRNT1 | TTC37 | TTC7A | TYK2 | UNC119 | UNC13D | UNC93B1 | UNG | USB1 | VPS13B |
| VPS45 | WAS | WIPF1 | XIAP | ZAP70 |  |  |  |  |  |  |  |

**Table S2. The information of primers for Sanger sequencing**

| Exons | Forward primers | Reverse primers | Length of PCR products | Tm |
| --- | --- | --- | --- | --- |
| MEFV-E1 | TCAGTTCCCACCAAGACACA | CTGAGACTCCCAATCCCCAG | 529bp | 59℃ |
| MEFV-E2 | GCCCGTTGTTTTCCTCAATTTC | CCTCCAGCAATCCTCCCG | 874bp | 59℃ |
| MEFV-E3 | AAGCTAGGAAGTGGGCAGAG | AATGCACCAACAACCCAGAG | 582bp | 59℃ |
| MEFV-E4 | GCCATTCCTCCCTCCTCTTT | CTGCTGGTTACCCTCTGTCC | 396bp | 59℃ |
| MEFV-E5 | AAGCCCACCTCTTATCCACC | CTTCACCCACTTGTTCCAGC | 439bp | 59℃ |
| MEFV-E6 | TGCTCCACTTCCACTGACAC | CTGACCAGATGCCCTTCTCC | 354bp | 60℃ |
| MEFV-E7, 8 | GACTTTTCTTTGTGTAGCTCAGG | CCTTTCTCCTACCTTTGCTCC | 594bp | 58℃ |
| MEFV-E9 | AGGAGAAAGGTCATGGCAGG | CAGAGAAGATGAGGTTGGGGT | 490bp | 59℃ |
| MEFV-E9, 10 | TGTTCCCTTGTGCTGTTGAC | CAACCTCCACCTCCCAGTAA | 700bp | 59℃ |
| MEFV-E10 | ATCCCTGTGCTCTCCCCTA | TGCTATAATCGGGTAGGCTCC | 832bp | 59℃ |

PCR=polymerase chain reaction; Tm=melting temperature.

**Methods for Meta-analysis**

The association between *MEFV* mutations and risk of SJIA was assessed with odds ratios (ORs) and 95% confidence intervals (95% CI). A comprehensive literature search was performed in the PubMed, EMBASE, and Web of Science databases up to the date of June 13, 2018. The keywords used for search were (“still’s disease” OR SJIA OR “systemic juvenile idiopathic arthritis” OR “adult onset still’s disease” OR AOSD) and *MEFV*. A cross-reference search of eligible articles was performed to identify studies not found in the computerized search. Case control studies written in English were included in the meta-analysis.

Further analysis was conducted with STATA 14.0 software (Stata Corp.; College Station, Texas, USA). Heterogeneity between studies was estimated with the Q-test (statistically significant heterogeneity existed when the P value<0.10) and the I^2^ statistic (25%, 50% and 75% were regarded as the cut-off points for low, moderate and high heterogeneity, respectively). A fixed-effects model (the Mantel-Haenszel method) was employed when I^2^<25%, otherwise a random-effects model (the Mantel-Haenszel method) was used. Begg’s funnel plots and Egger’s test were applied to assess the risk of publication bias, and the P value was set at 0.10. P values<0.05 were considered statistically significant.
