# Supplementary material for: The association of MEFV gene mutations with the disease risk and severity of systemic juvenile idiopathic arthritis
Source: Pediatr Rheumatol Online J. 2020 May 12;18:38. doi: 10.1186/s12969-020-00427-8 (PMC7218505; doi:10.1186/s12969-020-00427-8)
Supplement: Supplementary file 2 — Additional file 2. Supplemental results. [file 12969_2020_427_MOESM2_ESM.docx]

**Table S3. General Characteristics of the Studies Included in Meta-analysis**

| **Author** | **Publication Year** | **Country** | **Number**  **of cases** | **Number**  **of HC** | **Mutation studied** | **Genotyped method** |
| --- | --- | --- | --- | --- | --- | --- |
| Ayaz NA [14] | 2009 | Turkey | 35 | 100 | E148Q, M680I, M694V/I, V726A | reverse hybridization assay |
| Lotfy HM [15] | 2014 | Egypt | 54 | 30 | E148Q, M680I, M694V/I, V726A | reverse hybridization assay |
| Comak E[16] | 2013 | Turkey | 12 | 100 | E148Q, M680I, M694V/I, V726A | direct sequencing |
| Nonaka F[17] | 2014 | Japan | 49 | 105 | E148Q, M694V/I, | direct sequencing |
| Cosan F[18] | 2012 | Turkey | 20 | 103 | E148Q, M680I, M694V/I, V726A | PCR-RFLP |
| Kim JJ[19] | 2013 | Korea | 96 | 165 | E148Q | PCR-RFLP |
| the current study | 2018 | China | 57 | 2573 | E148Q | NGS + direct sequencing |

HC=healthy controls; PCR-RFLP=polymerase chain reaction restriction fragment length polymorphism; NGS=next generation sequencing.

**Table S4. Detailed Data for meta-analysis**

| Author | Year | Number of Case | Number of Control | M694V/I | | | | M680I | | | | V726A | | | | E148Q | | | |
| --- | --- | --- | --- | --- | --- | --- | --- | --- | --- | --- | --- | --- | --- | --- | --- | --- | --- | --- | --- |
|  |  |  |  | a | c | b | d | a | c | b | d | a | c | b | d | a | c | b | d |
| Ayaz NA | 2009 | 35 | 100 | 7 | 63 | 3 | 197 | 1 | 69 | 5 | 195 | 2 | 68 | 2 | 198 | 3 | 67 | 12 | 188 |
| Lotfy HM | 2014 | 54 | 30 | 4 | 104 | 0 | 60 | 5 | 103 | 1 | 59 | 17 | 91 | 0 | 60 | 8 | 100 | 4 | 56 |
| Comak E | 2013 | 12 | 100 | 4 | 20 | 3 | 197 | 0 | 24 | 5 | 195 | 0 | 24 | 2 | 198 | 2 | 22 | 12 | 188 |
| Nonaka F | 2014 | 49 | 105 | 2 | 96 | 0 | 210 | 0 | 98 | 0 | 210 | 0 | 98 | 0 | 210 | 29 | 69 | 52 | 158 |
| Cosan F | 2012 | 20 | 103 | 1 | 39 | 3 | 203 | 1 | 39 | 1 | 205 | 0 | 40 | 2 | 204 | 1 | 39 | 2 | 204 |
| Kim JJ | 2013 | 96 | 165 | 0 | 192 | 0 | 330 | 0 | 192 | 0 | 330 | 0 | 192 | 0 | 330 | 49 | 143 | 83 | 247 |
| the current study | 2018 | 55 | 2573 | 0 | 114 | 0 | 5146 | 0 | 114 | 0 | 5146 | 0 | 114 | 0 | 5146 | 26 | 88 | 1326 | 3820 |

a: the frequency of mutation allele in SJIA patients

b: the frequency of wide type allele in SJIA patients

c: the frequency of mutation allele in controls

d: the frequency of mutation allele in controls

**Begg’s funnel plots.**


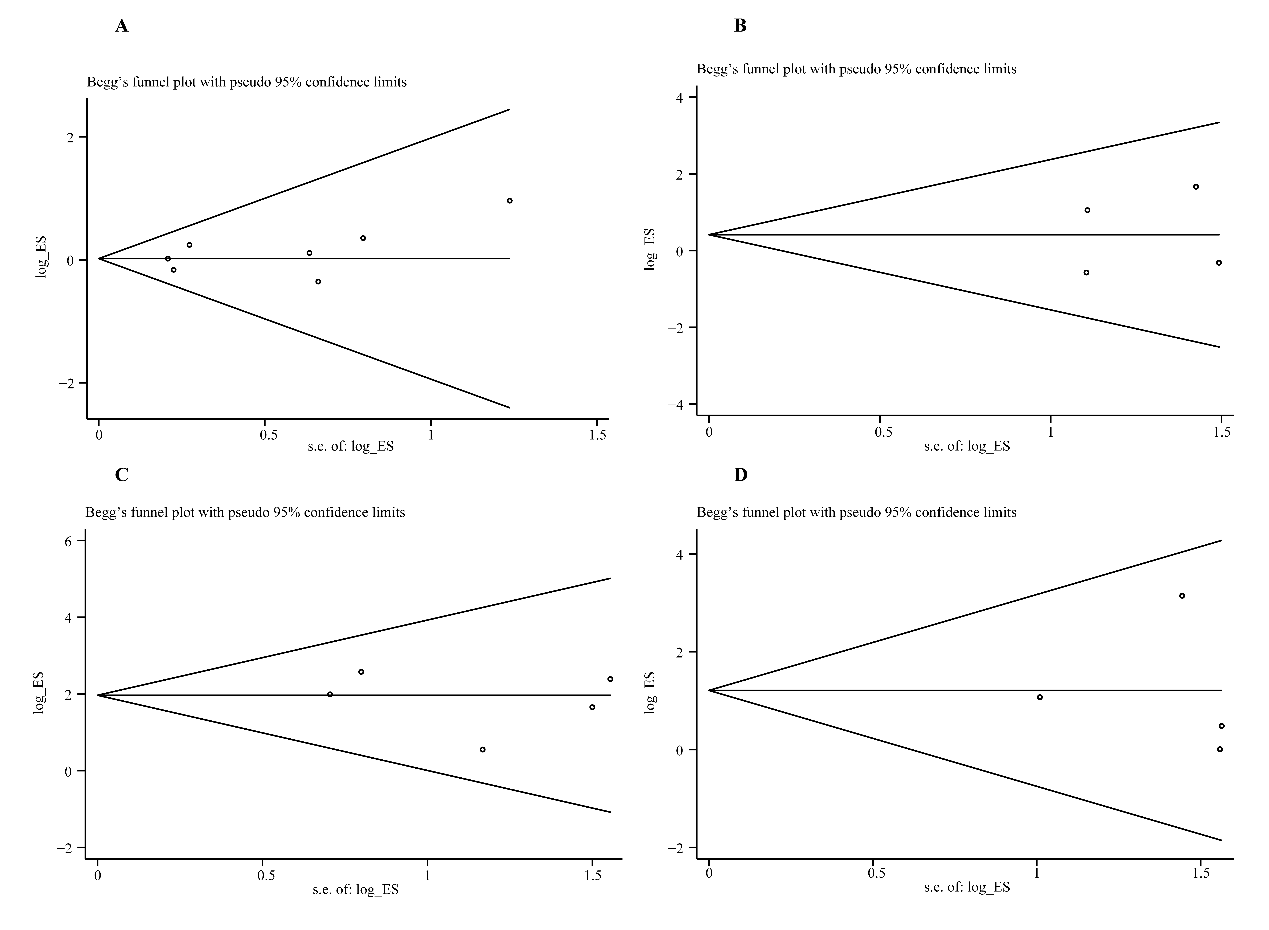


(A) E148Q; (B) M680I; (C) M694V/I; (4) V726A. No significant asymmetry was discovered in these plots.
